# Metabolite variation in the lettuce gene pool: towards healthier crop varieties and food

**DOI:** 10.1007/s11306-018-1443-8

**Published:** 2018-10-29

**Authors:** Rob van Treuren, Henriette D. L. M. van Eekelen, Ron Wehrens, Ric C. H. de Vos

**Affiliations:** 1 Centre for Genetic Resources, the Netherlands, Wageningen Plant Research, P.O. Box 16, 6700 AA Wageningen, The Netherlands; 2 Bioscience, Wageningen Plant Research, Droevendaalsesteeg 1, 6708 PB Wageningen, The Netherlands; 3 Biometris, Wageningen Plant Research, Droevendaalsesteeg 1, 6708 PB Wageningen, The Netherlands

**Keywords:** Crop improvement, Genetic resources, Lettuce, Untargeted metabolomics, Phytochemical variation, Vitamin C

## Abstract

**Introduction:**

Lettuce (*Lactuca sativa* L.) is generally not specifically acknowledged for its taste and nutritional value, while its cultivation suffers from limited resistance against several pests and diseases. Such key traits are known to be largely dependent on the ability of varieties to produce specific phytochemicals.

**Objectives:**

We aimed to identify promising genetic resources for the improvement of phytochemical composition of lettuce varieties.

**Methods:**

Phytochemical variation was investigated using 150 *Lactuca* genebank accessions, comprising a core set of the lettuce gene pool, and resulting data were related to available phenotypic information.

**Results:**

A hierarchical cluster analysis of the variation in relative abundance of 2026 phytochemicals, revealed by untargeted metabolic profiling, strongly resembled the known lettuce gene pool structure, indicating that the observed variation was to a large extent genetically determined. Many phytochemicals appeared species-specific, of which several are generally related to traits that are associated with plant health or nutritional value. For a large number of phytochemicals the relative abundance was either positively or negatively correlated with available phenotypic data on resistances against pests and diseases, indicating their potential role in plant resistance. Particularly the more primitive lettuces and the closely related wild relatives showed high levels of (poly)phenols and vitamin C, thus representing potential genetic resources for improving nutritional traits in modern crop types.

**Conclusion:**

Our large-scale analysis of phytochemical variation is unprecedented in lettuce and demonstrated the ample availability of suitable genetic resources for the development of improved lettuce varieties with higher nutritional quality and more sustainable production.

**Electronic supplementary material:**

The online version of this article (10.1007/s11306-018-1443-8) contains supplementary material, which is available to authorized users.

## Introduction

Lettuce (*Lactuca sativa* L.) is a main leafy vegetable for human consumption and an economically important food crop worldwide. In 2014 over 1 million hectares were harvested for chicory and lettuce combined, with a total production of nearly 25 million tonnes (FAOSTAT 2017). Particularly in the United States and Europe, new lettuce varieties are regularly introduced to the market. For example, the number of new varieties included in the European Common Catalogue usually exceeds 100 per year (Van Treuren et al. 2008).

A wide variety of crop types can be distinguished among current lettuce cultivars (Lebeda et al. 2007). Crispheads and butterheads are heading lettuces that are most common in the United States and Western Europe, respectively. Cos lettuces form tall loose heads and are mostly cultivated in the Mediterranean area. Also latin lettuces form loose heads and are popular in the Mediterranean area, while thick leathery leaves are characteristic for this crop type. Cutting lettuces, which are popular in both the United States and Europe, are non-heading and show a large variation in leaf morphology. Stalk lettuces are mainly cultivated in China and Egypt for the consumption of their long and thickened stem. Oilseed lettuces are mainly grown in Egypt for cooking purposes due to the high oil content of the seeds. Oilseed lettuce largely resembles *Lactuca serriola* L., a wild relative that is generally considered the most likely origin of cultivated lettuce. Therefore, oilseed lettuce is considered the most primitive form in the domestication of lettuce. Also stem lettuce is thought to have arisen early in the domestication history, and is considered the predecessor of the cos lettuces that in their turn are seen as the source from which the crispheads, butterheads, cutting and latin lettuces have evolved (Ryder 1999).

Crop-related species are often classified according to the gene pool concept, which is based on the level of inter-fertility with the crop species. The primary gene pool consists of the crop species and those wild relatives with which it can easily be inter-crossed. Species that can produce at least some fertile hybrids are assigned to the secondary gene pool, while species that can only be crossed through specific techniques, such as embryo rescue or bridge crosses, are considered to belong to the tertiary gene pool (Harlan and de Wet 1971). The genus *Lactuca* consists of more than 100 species, of which 20 are considered to belong to the lettuce gene pool (Van Treuren et al. 2012). *L. sativa* and *L. serriola* are the main species of the primary lettuce gene pool that further consists of *L. aculeata, L. altaica, L. azerbaijanica, L. dregeana, L. georgica* and *L. scarioloides*. The secondary gene pool is formed by *L. saligna* alone, although the position of *L. virosa* has been debated, either representing a secondary or tertiary gene pool species. The tertiary gene pool includes *L. acanthifolia, L. aurea, L. longidentata, L. orientalis, L. quercina, L. sibirica, L. taraxacifolia, L. tatarica, L. viminea* and *L. watsoniana*. Despite their importance for plant breeding, wild relatives of lettuce are generally underrepresented in genetic resources collections, with the exception of *L. serriola*, and to some extent of *L. saligna* and *L. virosa* (Van Treuren et al. 2012). These three species have been the most widely used wild relatives for the improvement of lettuce cultivars (Lebeda et al. 2014).

As in many crops, breeding targets in lettuce include resistances against pests and diseases, of which lettuce downy mildew and lettuce aphids represent the most significant threats to crop production (Parra et al. 2016; Walley et al. 2017). Lettuce breeding is also directed to consumer quality characters, such as shelf life, leaf shape and colour, taste and nutritional value (Still 2007; Mou 2008; Davey and Anthony 2011). In general, many key crop traits are known to be directly or indirectly related to metabolite composition, and lettuce has been investigated for a variety of specific metabolites related to product quality (e.g. Oh et al. 2009; Chadwick et al. 2016; Kim et al. 2016). Sensitive mass spectrometry-based analytical techniques, and especially untargeted approaches including unbiased data processing, nowadays enable the analysis of up to several hundred metabolites in parallel, providing deep insight into the metabolome similarities and differences between series of samples, e.g. in relation to phenotypic or genotypic differences (Khan et al. 2012; Wahyuni et al. 2013). Metabolomics can be regarded as a deep phytochemical phenotyping instrument and a potential powerful tool in the exploitation of genetic resources in plant breeding (Keurentjes et al. 2006; Fernie and Schauer 2009; Zhu et al. 2018). Liquid chromatography coupled to high resolution mass spectrometry, herein referred to as LCMS, is the most preferred platform for detecting a large variety of non-volatile semi-polar secondary metabolites, including major biochemical classes like phenolic acids, phenylpropanoids, flavonoids, alkaloids, saponins, glucosinolates and sesquiterpenes, next to many other, yet unknown compounds (De Vos et al. 2007; Iijima et al. 2008; García et al. 2017). For lettuce, large-scale or essentially untargeted LCMS-based metabolomics approaches have previously been used to investigate metabolite differences among babyleaf, romaine and iceberg cultivar types (Abu-Reidah et al. 2013) and between green and red oakleaf lettuce (Viacava et al. 2017), as well as to obtain insight in metabolites related to browning of fresh-cut romaine lettuce (García et al. 2017). However, comprehensive metabolomics studies of the lettuce gene pool are yet non-existing.

The lettuce genome has recently been published (Reyes-Chin-Wo et al. 2017), while reference genomes of *L. serriola, L. saligna* and *L. virosa* are expected to become publically available in the near future and large-scale DNA resequencing projects are underway for *Lactuca* germplasm. The wealth of DNA sequencing data is expected to open the door for functional genomics approaches, ultimately leading to more efficient plant breeding towards healthier lettuce varieties for more sustainable production and with improved nutritional quality for consumers. However, such approaches can only be successful in the presence of comprehensive, high-quality phenotypic information (Still 2007).

Here we used an LCMS-based large-scale and unbiased metabolite profiling approach in order to deeply phytochemically phenotype 150 different *Lactuca* genotypes, including cultivated lettuce and its wild relatives. These genotypes were selected from the lettuce collection of the Centre for Genetic Resources, the Netherlands (CGN), which currently is the largest in the world and which has a relatively high representation of wild relatives. The accessions have been relatively well characterized by morphological and molecular descriptors, while also many quality trait data have been collected, including nearly 40,000 data points on downy mildew resistance alone (CGN 2017a). This comprehensive analysis of phytochemical variation, coupled with trait variation, is unprecedented for *Lactuca* germplasm. Specifically, our study aimed to identify promising phytochemical genetic resources for crop improvement.

## Methods

### Study material

Research lines for the present study were chosen from a group of nearly 500 *ex situ* accessions selected for DNA re-sequencing within the context of the project entitled ‘International *Lactuca* Genomics Consortium’ (ILGC). To obtain a test panel capturing a wide diversity of the lettuce gene pool, the accessions selected within the ILGC represented a core set from the CGN lettuce collection, supplemented with materials from other organizations. This core set mainly covered all seven crop types of cultivated lettuce and the main wild relatives *L. serriola, L. saligna* and *L. virosa*. The core set was supplemented with specimens of less-well studied wild *Lactuca* species (Van Treuren et al. 2012). Main criteria to select accessions within taxonomic groups included variation in previously collected phenotypic (e.g. Van Treuren et al. 2013) and genotypic (e.g. Van Hintum 2003) data. For wild species, also variation in geographic origin was taken into account. A subset of 150 accessions was chosen for the present study following the same selection procedures as used for the core set, provided that species were represented with at least two accessions in order to enable examination of intra-specific variation (Supplementary Table 1).

A single plant of each accession of the core set was grown for tissue sampling and seed production in the greenhouse facilities of Wageningen University in 2016, following the procedures outlined on CGN’s website (CGN 2017a). The selected set of 150 accessions used in the present study were sampled on May 24, when the cultivated lettuces had reached maturity and the wild species were close to bolting. Per genotype, three to six leaves, depending on their size, were collected and immediately frozen in liquid nitrogen. All samples were maintained at − 80 °C awaiting further processing for metabolite analysis. All plants of the core set, with the exception of the outcrossing species, were maintained for seed production by self-fertilization. Samples of successful seed multiplications were stored at CGN as a special collection (Van Treuren and Van Hintum 2014) of Single Seed Descent lines (CGN 2017b).

### Metabolite profiling

Frozen leaf material was ground into a fine powder using liquid nitrogen. Material for quality control samples (technical repeats) was prepared by pooling an equal aliquot of the frozen powder of each sample. This sample pool was subsequently treated similarly and simultaneously with the 150 individual samples.

For LCMS-based profiling, 300 mg fresh weight of each plant powder was extracted as described previously (De Vos et al. 2007) by adding 900 µl of 99.87% methanol (MeOH) containing 0.13% formic acid (FA), which resulted in a final concentration of about 75% MeOH and 0.1% FA assuming a leaf water content of about 95%. Frozen samples were vigorously vortexed immediately after adding the MeOH–FA solution to the frozen material, sonicated for 15 min and centrifuged at maximum speed for 15 min. From each sample 500 µl of the supernatant was transferred via a 0.45 µm PTFE filter into 700 µl glass inserts in a 96-wells deep well block. For every 15 samples, 6 quality control samples were simultaneously extracted. The compounds present in these aqueous-methanol extracts were separated on a Luna C18 column (2.0 × 150 mm, 3 µm; Phenomenex) at 40 °C, using an Acquity HPLC module (Waters) to apply a 45 min linear gradient of 5–75% acetonitrile in 0.1% FA in water at a flow rate of 0.19 ml/min. Both a photodiode array (PDA) detector to record UV/Vis-light absorbance spectra in the range of 220–700 nm and an LTQ-Orbitrap FTMS hybrid system (Thermo) operating in negative electrospray ionization mode was used for metabolite detection (Van der Hooft et al. 2012). Mass profiles were collected in the m/z range 95–1300 in centroid mode and at a resolution of 60,000.

For extraction of ascorbic acid (vitamin C), 300 mg frozen leaf powder was mixed with 1.2 ml of ice cold extraction solvent consisting of 5% meta-phosphoric acid in water containing 1 mM diethylene penta-acetic acid. Samples were vortexed, sonicated for 15 min and centrifuged at maximum speed for 20 min. The supernatant was filtered over an 13 mm nylon-66 MDI filter, with a pore size of 0.2 µm and transferred to an 1.8 ml HPLC vial. Ascorbic acid was analysed using a Waters W2695 HPLC module connected to a Waters W2996 PDA detector (220–400 nm). Ascorbic acid was separated using a YMC-Pro C18 3.9 × 150 mm column (YMC corporation, Japan), 50 mM potassium-phosphate buffer pH 4.4 (eluent A) at 30 °C and a flow rate of 0.5 ml/min. Ascorbic acid eluted at a retention time of 5.2 min and its peak area was determined at 262 nm. A calibration series of ascorbic acid in extraction solution, ranging from 2.5 to 300 µg/ml, was prepared for quantification purposes.

### Data analysis

#### LCMS data processing

The raw LCMS data were processed using Metalign software (Lommen 2009; http://www.metalign.nl) for baseline correction, noise estimation, and ion-wise mass spectral alignment. This resulted in 310,262 individual mass signals. After removing low and inconsistent signals, i.e. present in < 3 samples or with an ion intensity lower than 1000 in all samples, 55,243 mass signals remained that were subjected to MSClust software (Tikunov et al. 2012) in order to assemble redundant mass signals derived from the same metabolite, including natural isotopes and unintended but unavoidable adducts and in-source fragments, based on their corresponding retention time and variation across samples. This resulted in the relative peak intensities of 2026 mass signal clusters each representing a (reconstructed) putative metabolite present in at least two plants.

Using multiple online databases, including KNApSAcK (http://kanaya.naist.jp/KNApSAcK/), Dictionary of Natural Products (http://dnp.chemnetbase.com), Metlin (https://metlin.scripps.edu/), HMD (http://www.hmdb.ca), in-house libraries based on standards, as well as the mass spectra information within the clustered mass peaks and from additional LCMS runs (actually LC–PDA–LTQ–FTMS) generating accurate mass spectral trees from the top 3 intensity ions every 30 s (Van der Hooft et al. 2012), selected metabolites were manually annotated as far as was possible with the mass data and the UV/Vis-absorbance spectral data obtained.

#### Statistical methods

During the experiment it appeared that the LCMS profile of one of the *L. aculeata* samples (TKI-464) strongly resembled those obtained for the four *L. georgica* samples. Re-analysis of the original leaf material indicated that this finding was clearly due to a leaf sampling error, and therefore all data of TKI-464 were discarded from further statistical analyses. Due to experimental failure, vitamin C data were absent for both TKI-454 and TKI-471.

Metabolites detected by LCMS were subjected to multivariate analysis, i.e. Hierarchical Clustering Analysis, using GeneMaths XT software version 2.12 (Applied Maths, Belgium). Metabolite intensity data were firstly log2-transformed and then mean-centred.

LCMS results were also analysed separately for *L. sativa* and its main wild relatives *L. serriola, L. saligna* and *L. virosa*. The number of metabolites detected in these four species and the overlap in phytochemical composition was represented by a Venn diagram constructed in MS-Excel. To identify metabolites that are more or less species-specific among these four species, a selection was made of the phytochemicals detected in at least 95% of the samples per species. Phytochemicals were then ranked according to the highest ratio between the average metabolite level in one specific species and the highest level among the other three species. Statistical analyses were performed in R (R Core Team 2017) using in-house scripts.

Metabolite data were related to trait data, such as resistances to pests and diseases, previously collected for accessions of the CGN lettuce collection (CGN 2017c) using random forests (Breiman 2001), as implemented in the R package RandomForest (Liaw and Wiener 2002). Briefly, this nonlinear modeling technique creates an ensemble of regression trees, each tree receiving only a subset of the data, both in terms of sample and variables. The final prediction is then a combination of the predictions of the individual trees. The technique is appealing because it usually requires no tuning, and automatically returns cross-validated prediction errors. Variable importance is assessed by observing the change in prediction error upon removing a variable, leading to an increase in prediction error in case of important variables.

## Results

### Untargeted metabolite profiling

Natural variation in phytochemicals was determined in 150 genotypes of cultivated and wild *Lactuca* species by preparing crude leaf extracts in 75% acidified methanol and an untargeted metabolite profiling approach using HPLC with Orbitrap FTMS at high mass resolution in negative ionization mode. Samples were prepared in two batches of 75 randomly chosen genotypes each. Six quality control samples (QCs) were included per batch and equally divided over the study samples. In order to prevent possible batch effects in the LCMS-analysis, all samples were randomly analysed in a single series. The total analysis time was 162 h without interruption. Subsequent untargeted data processing using the Metalign-MSClust based workflow resulted in relative intensity data for 2026 putative metabolites across all genotypes (Supplementary Table 2). For those 851 compounds detected in all 12 QC samples the average technical variation was 11.8%, including sample weighing, extraction, LCMS analysis and untargeted data processing. Based on the detected accurate mass of the putative molecular ions, and for selected metabolites also their MSn fragmentation data, we could tentatively annotate a series of amino acids, organic acids, phenylpropanoids, flavonoids, anthocyanins and sesquiterpene lactones among the 2026 metabolites.

### Phytochemical variation within the genus *Lactuca*

**Fig. 1:**
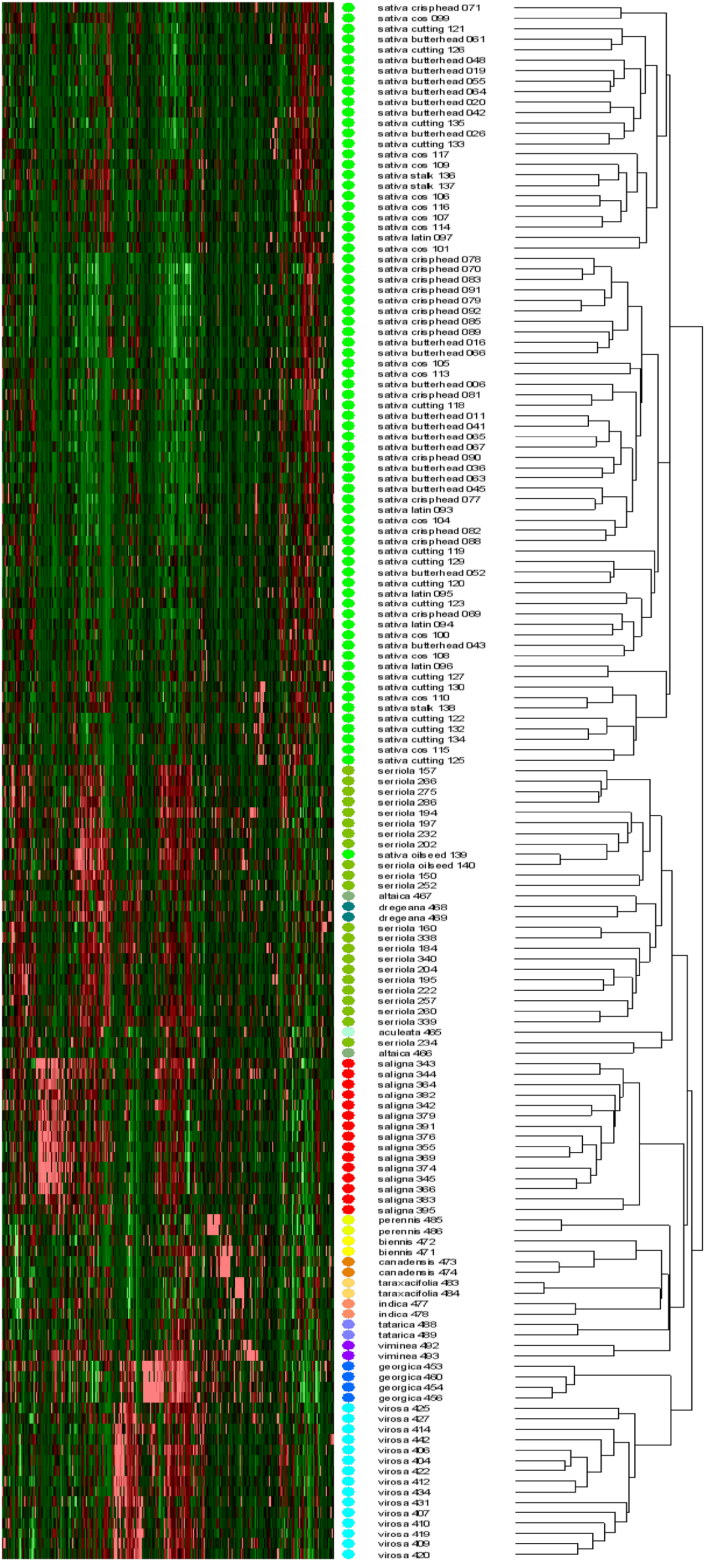
Hierarchical cluster analysis of the research lines, denoted by taxonomic group and line identifier, based on 2026 metabolites detected by LCMS

A hierarchical cluster analysis of the genotypes based on all detected metabolites was largely in line with the presumed taxonomic relationships (Fig. 1). With the exception of TKI-139, all *L. sativa* samples clustered in a single group, separate from the wild *Lactuca* species. This TKI-139 is documented as cultivated oilseed lettuce and appeared phytochemically closely related to TKI-140, a specimen of *L. serriola* also documented as oilseed lettuce. Within the *L. sativa* group no clear clustering according to crop type was observed. All plants of the primary gene pool species *L. aculeata, L. altaica, L. dregeana* and *L. serriola* clustered together in a single group, while the plants of *L. georgica*, also considered to belong to the lettuce primary gene pool, showed a closer relationship to those of *L. virosa*, a species regarded to belong to the secondary or tertiary gene pool. All samples of *L. saligna*, representing the secondary gene pool, clustered together in a single group. Also the more exotic species *L. biennis, L. canadensis, L. indica, L. perennis, L. taraxacifolia, L. tatarica* and *L. viminea*, representing tertiary gene pool species or even taxa considered more distantly related to cultivated lettuce, formed a separate group within the wild *Lactuca* germplasm. The finding that the observed genotype clustering patterns were largely in line with taxonomic relationships indicate that the variation in phytochemical composition is to a large extent genetically determined, suggesting excellent possibilities for breeding towards new lettuce varieties with improved trait characteristics.

**Fig. 2:**
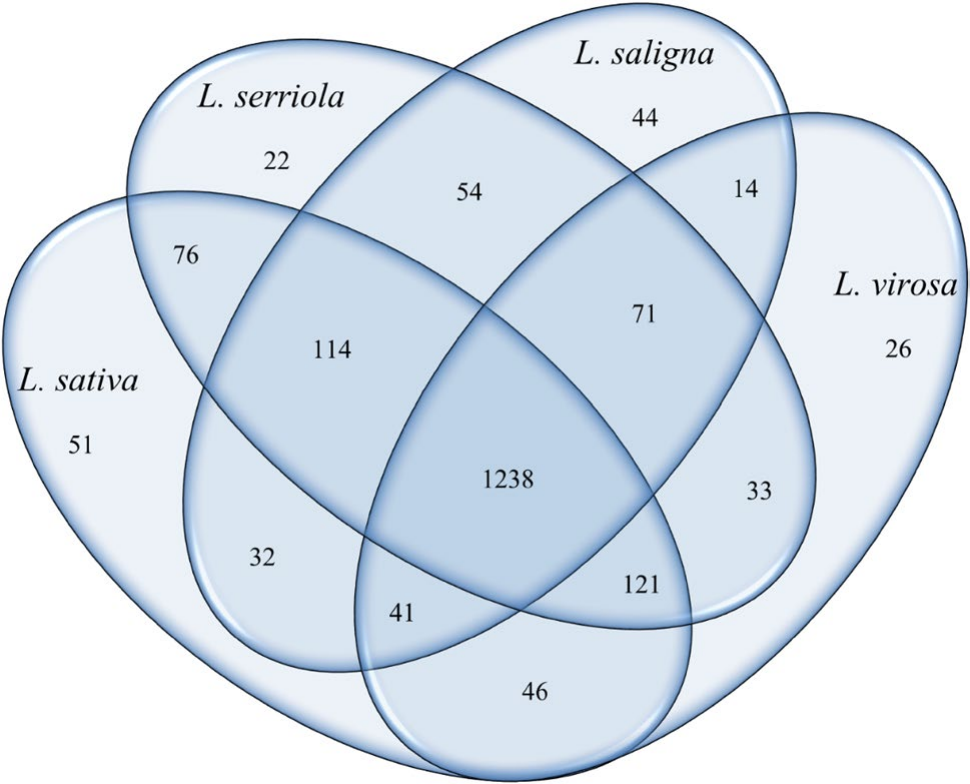
Venn diagram for *L. sativa, L. serriola, L. saligna* and *L. virosa* showing the extent of overlap in phytochemical composition

**Fig. 3:**
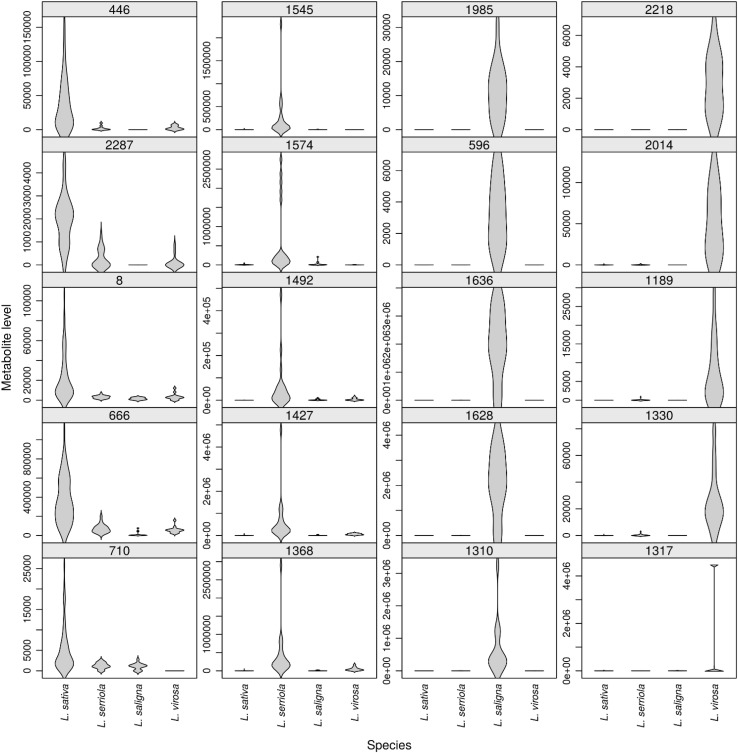
Violin plots of the five most specific phytochemicals for each of the species *L. sativa, L. serriola, L. saligna* and *L. virosa*. Each plot also shows the LCMS data of the other three species. Phytochemicals are denoted by their cluster identification code at the top of each plot

A separate exploration of the LCMS data was performed for *L. sativa* and its three wild relatives most commonly used in plant breeding, i.e. *L. serriola, L. saligna* and *L. virosa*, in order to determine phytochemical overlap and to identify compounds most characteristic to one of these species. For this analysis TKI-139 and TKI-140 were disregarded, as these oilseed samples were found to represent outliers within the *L. sativa* group (results not shown). Out of the total of 2026 phytochemicals, 43 (2.1%) were not detected in any genotype of *L. sativa, L. serriola, L. saligna* and *L. virosa*, while 1238 (61.1%) were observed in each of the four species (Fig. 2). The number of phytochemicals observed in only a single species ranged from 22 in *L. serriola* to 51 in *L. sativa*. Out of the 1932 phytochemicals detected in either of the wild species *L. serriola, L. saligna* or *L. virosa*, 264 (13.7%) were not observed in *L. sativa*. The strong variation in phytochemical composition among these four species was also evident from phytochemicals that were more or less species-specific (Fig. 3). Ratios between the average intensity value of *L. sativa* and the highest average value among the other three species ranged from 14.4 (ID 446: unknown compound) to 4.1 (ID 710: putatively identified as 3,4-dihydroxy-E-cinnamoyl-altro-heptulose) for the presented *L. sativa* phytochemicals. For *L. serriola* metabolites these abundance ratios ranged from 268.3 (ID 1545: dicoumaroyl tartaric acid) to 8.8 (ID 1368: coumaroyl–caffeoyl-malic acid). More than 1000-fold higher average intensity values were detected for the more specific phytochemicals of *L. saligna* when compared to the other three species, and more than 100-fold for *L. virosa* (Fig. 3). The selected metabolites are highlighted in the biplot shown in Supplementary Fig. 1, confirming their relatively high specificity among the four species.

As shown in Fig. 3, phytochemical levels may vary strongly within a single *Lactuca* species, indicative of intra-specific genetic variation. Phytochemicals more or less specific to a species included phenylpropanoids like caffeoylmalic acid (ID 666) in *L. sativa*, two isomers of coumaroyl–caffeoylmalic acid (ID 1427 and ID 1368) in *L. serriola* and phenylethanol-glycoside (ID 596) in *L. saligna*, as well as the sesquiterpene-lactone lactucopicriside (ID 1317) and the flavone methoxyluteolin-hexoside (ID 1189) in *L. virosa*.

### Phytochemical variation and plant health

**Fig. 4:**
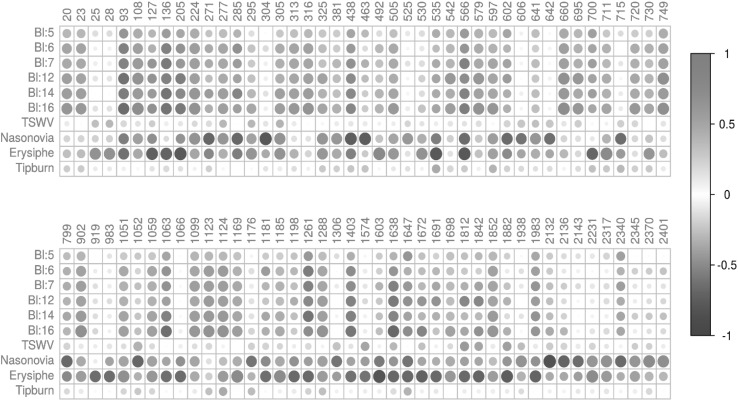
Correlation between the relative intensity of the phytochemicals detected by LCMS and the level of resistance against each of the pathotypes Bl:5, Bl:6, Bl:7, Bl:12, Bl:14 and Bl:16 of downy mildew (*Bremia lactucae*), tomato spotted wilt virus (TSWV), lettuce aphid *Nasonovia ribisnigri*, powdery mildew (*Erysiphe cichoracearum*) and tipburn. Only phytochemicals with at least one correlation higher than 0.6 or lower than − 0.6 are presented

Correlations between phytochemicals and resistance traits are presented in Fig. 4, showing only those compounds for which at least one correlation higher than 0.6 in absolute value was observed. Clearly, several groups of phytochemicals can be defined showing similar correlations with resistance traits. These findings may point towards the influence of population structure, causing spurious relationships between different characters, but may also reflect related phytochemicals derived from the same biochemical pathway. Therefore, whether the observed correlations are based on causal relationships remains subject for further study.

**Fig. 5:**
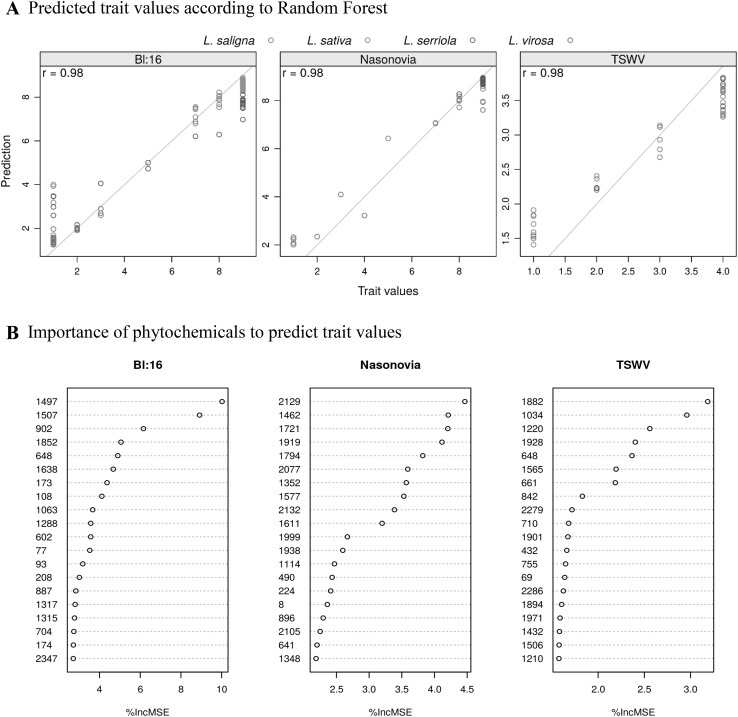
**a** Observed and predicted values for resistance against downy mildew Bl:16, *Nasonovia ribisnigri* and TSWV. Predictions are based on the LCMS data using a Random Forest model. A perfect correlation is indicated by the grey line. The correlation coefficient is presented in the top left corner of each graph. Data points for each of the species *L. sativa, L. serriola, L. saligna* and *L. virosa* are colour-marked. **b** Importance of phytochemicals to predict trait values. The importance is expressed as %IncMSE, representing the extent of deterioration of the model when the phytochemical is discarded. Only the 20 most important phytochemicals for each of the traits are presented

Figure 5a shows predicted resistance values for three of the resistance traits obtained from random forest models using the metabolite abundance data. In each of these cases the measured and predicted trait values were found to be highly correlated, suggesting that metabolites play a key role in these traits. Inspection of the models leads to estimates of the importance of each phytochemical in the models, the top 20 of which is visualized in Fig. 5b for all three models. Some of these phytochemicals could be annotated based on available MS data, such as the most important metabolite (ID 1497) in the prediction of resistance against downy mildew pathotype Bl:16, which was putatively identified as the sesquiterpene-lactone jacquinelin (11,13-dihydro-8-deoxylactucin) previously reported for *L serriola* and *L virosa* (http://kanaya.naist.jp/KNApSAcK). The second most important compound in this model (ID 1507) was also a sesquiterpene-lactone, putatively identified as melampodin B, suggesting an important role for this specific class of compounds in downy mildew resistance.

### Phytochemical variation and consumption-related traits

The LCMS data revealed considerable genetic variation in known lettuce phenolic compounds that are generally considered beneficial to human health, such as the phenylpropanoids caftaric acid (ID 343: 399 fold difference between the highest and lowest relative abundance value), chlorogenic acid (ID 415: 36.5 fold), chicoric acid (ID 1090: 109 fold) and the flavonoid quercetin-glucuronide (ID 852: nearly 3000 fold), of which the highest relative abundance values were detected in *L. georgica* (Supplementary Table 2). Sesquiterpene-lactones belong to a class of phytochemicals known to influence the bitterness of lettuce and other leafy vegetables of the Asteraceae family (Sessa et al. 2000) and also within this class considerable variation was detected within and between the examined *Lactuca* species. For instance, the bitter compound deoxylactucin-sulphate (ID 1532) was relatively high in *L. virosa, L. dregeana* and *L. tatarica*. Jacquinelin (11,13-dihydro-8-deoxylactucin; ID 1497), a sesquiterpene-lactone related to bitterness in chicory (Testone et al. 2016), was present in all accessions of *L. saligna*, in 2 out of the 21 *L. serriola* accessions and in both *L. perennis* accessions (Supplementary Table 2). With regard to red leaf colour, cyanidin-malonyl-glucoside (ID 484) was the main compound, based on its peak area of the HPLC-DAD profiles at 510 nm, and was detected in specific *L. sativa* accessions of cos, cutting and stalk lettuce, as well as in several accessions of wild species (Supplementary Table 2).

**Fig. 6:**
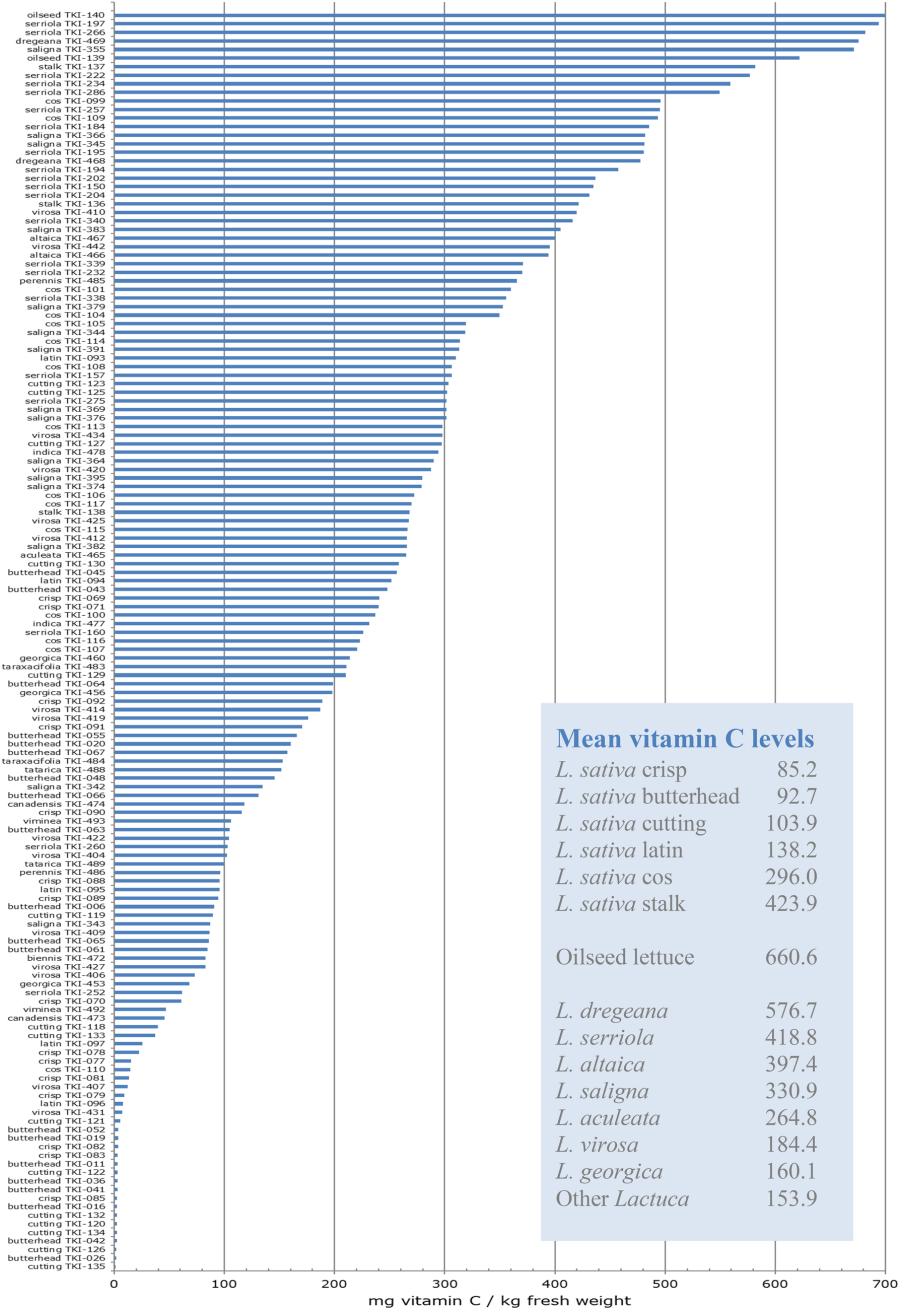
Vitamin C content in mg per kg fresh weight of the research lines, denoted by line identifier and taxonomic group

Since lettuce is regarded as a good source of vitamin C in the human diet, we separately extracted and quantified vitamin C (ascorbic acid) using a dedicated HPLC method. Vitamin C levels showed considerable variation both within and between the examined *Lactuca* species (Fig. 6). Relatively low values were found for the more modern crop types, their mean level ranging from 85.2 mg per kg fresh weight for crisphead to 138.2 for latin lettuce. Multiples of these mean values were observed for the more primitive crop types cos, stalk and oilseed lettuce. Relatively high vitamin C levels were also detected in the primary gene pool species *L. dregeana, L. serriola, L. altaica* and *L. aculeata*, and in the secondary gene pool species *L. saligna*. Mean value of the primary gene pool species *L. georgica* resembled that of *L. virosa*.

## Discussion

### Phytochemical variation in the lettuce gene pool

Lettuce is an economically important leafy vegetable crop with a worldwide human consumption. However, current lettuce cultivars are generally not specifically acknowledged for their taste and nutritional value, while their cultivation suffers from limited resistances against several pests and diseases. As such key traits largely depend on the presence of specific phytochemicals, a series of 150 different accessions from both cultivated lettuce and its wild relatives was screened for their variation in mainly secondary metabolites using an untargeted, accurate mass LCMS approach. The observed phytochemical variation was not distributed randomly, but showed a clear pattern that was largely in line with the presumed gene pool structure of lettuce. With the exception of TKI-139, all cultivated lettuce genotypes clustered together in a single group, separate from their wild relatives. The TKI-139 is registered as an oilseed lettuce and its species name has been changed from *L. serriola* into *L. sativa* in the past. Oilseed is the most primitive crop type in lettuce, possessing phenotypic characters that strongly resemble those of *L. serriola*. Therefore, oilseed lettuces are sometimes assigned to *L. sativa*, sometimes to *L. serriola* or sometimes considered as intermediate types between the two species. These taxonomic ambiguities have also been found in molecular studies (Koopman et al. 2001; Van Treuren and Van Hintum 2009). Within the group of *L. sativa*, no clear distinction between the crop types was observed. Also molecular studies have shown the absence of a clear population structure within *L. sativa*, which is likely due to the use of breeding parents from different crop types in breeding for new cultivars (Hu et al. 2005; Van Treuren et al. 2008; Simko 2009; Van Treuren and Van Hintum 2009). With the exception of *L. georgica* all wild species of the primary lettuce gene pool clustered together in a single group, separate from all other wild *Lactuca* species. Within this group of primary gene pool species, the analysed accessions of *L. serriola, L. altaica, L. dregeana* and *L. aculeata* did not cluster in distinct species groups. These fully interfertile species have been considered conspecific based on their high similarity at the molecular level (Koopman et al. 2001). The *L. georgica* accessions used in the present study were originally collected in the Trans Caucasus as *L. virosa*, but their taxonomic status was changed because of their strong phenotypic resemblance with more recently collected samples of *L. georgica* from the same geographical area and their strong differences in molecular variation in comparison with other accessions of *L. virosa* (CGN, unpublished). Nevertheless, the two species share several phenotypic features, such as the blackish seed colour. The two species also clustered together based on their leaf phytochemical composition. *L. georgica* is generally considered to be closely related to *L. serriola* based on morphological characters and is presumed (largely) interfertile with species from the primary gene pool (Zohary 1991). Considering its observed closer phytochemical relationship to *L. virosa* than to *L. sativa*, crossing experiments with cultivated lettuce are needed to test the presumed gene pool status of *L. georgica*, and hence their usefulness in plant breeding. Recently, a similar conclusion was drawn from a molecular marker study in *L. georgica* and related species (Beharav et al. 2018). Accessions of the secondary gene pool species *L. saligna*, those of the tertiary gene pool and those that are more distantly related to cultivated lettuce nicely clustered together according to species. The observed phytochemical relationships are largely in line with those based on molecular marker and DNA sequence data (Koopman et al. 1998, 2001; Wei et al. 2017) and hence indicate that the observed variation in phytochemicals is to a large extent genetically determined, providing excellent possibilities for breeding towards novel lettuce varieties with improved phytochemical composition.

### Phytochemicals and crop quality traits

A high level of species-specificity among the investigated research materials was shown by many phytochemicals, of which several could be related to traits that are associated to disease resistance, nutritional quality or both. For instance, we observed huge genetic variation in phytochemicals likely contributing to the widely accepted nutritional health benefits of fruits and vegetables, including a variety of phenylpropanoids such as chlorogenic acid and chicoric acid, and several flavonoids such as quercetin-conjugates. Since vitamin C is generally considered beneficial to human health, we specifically analysed this plant phytochemical. Our results revealed large variation in vitamin C both within and between *Lactuca* species. Relatively high vitamin C levels were observed in the more primitive lettuces and the closely related wild relatives, indicating their high potential to improve this health trait in the modern crop types crisp, butterhead, cutting and latin lettuce.

Although in the present study we did not quantify metabolite levels using authentic standards, the relative abundance value (chromatographic peak intensity) of each metabolite detected can be directly compared across all samples. According to the Phenol-Explorer database (Neveu et al. 2010), the level of chlorogenic acid in cultivated lettuce is about 4 mg/100 g FW. As in our study the relative intensity of the peak corresponding to chlorogenic acid (ID 415) was roughly 10 fold higher in *L. saligna* than in the average of lettuce cultivars (Supplementary Table 2), good resources are available in the lettuce gene pool to breed for new cultivars with significantly higher levels of this phenolic antioxidant. Significant variation was also observed for other phenolic antioxidants. Interestingly, in most *L. saligna* accessions both chicoric acid (di caffeoyl-tartaric acid; ID 1090) and caftaric acid (mono caffeoyl-tartaric acid; ID343) were relatively low compared to other species, while chlorogenic acid (caffeoyl-quinic acid) is relatively high, suggesting that the tartaric acid esterification to caffeic acid is limited in this particular species. In contrast, *L. georgica* contained relatively high levels of both chicoric and caftaric acid.

Sesquiterpene lactones (SLs) are present in leaves and stems of main Asteraceae crop plants such as lettuce, endive and chicory, and thus also part of the human diet. Several SLs including lactucin, 8-deoxylactucin and lactucopicrin are well-known for their bitter taste (e.g. Van Beek et al. 1990) and lettuce breeding has been directed towards relatively low levels of such bitter compounds. On the other hand, depending upon their exact chemical structure, SLs are potentially beneficial to human health by exerting relevant pharmacological activities including anti-inflammatory and anti-carcinogenic effects, while in the plant they function as anti-microbial or anti-feeding compounds helping to protect against pests and diseases (e.g. Chadwick et al. 2013). In the present study, among the most important compounds for the prediction model of resistance against downy mildew pathotype Bl:16 were SLs, putatively identified as Jacquinelin (11,13-dihydro-8-deoxylactucin; ID 1497) and melampodin B (ID 1507). Within both the primary and secondary gene pool of lettuce several wild accessions were identified having relative high levels of one or more specific SLs. As the bitterness perception of SLs as well as their health benefits for either or both plant and human likely depend on the exact chemical structure of the specific compound, this study provides unprecedented data of accessions that may be exploited in future research towards SL biosynthesis and improving either taste or health of lettuce cultivars.

## Conclusion

This unprecedented large-scale metabolomics study of a substantial collection of *Lactuca* samples revealed significant diversity in phytochemical composition within the lettuce gene pool, including variation for metabolites related to plant or human health. Large-scale DNA sequence data of the examined research materials are expected in the near future, after which the genomic information will be linked to information on plant traits and phytochemical variation (CGN 2017b). This will substantially improve our insight in the exact role of these detected phytochemicals in lettuce quality traits and genes involved in their biosynthesis. The identification of appropriate genetic resources and the availability of DNA markers will strongly contribute to an accelerated development of new lettuce varieties with higher nutritional quality and more sustainable production. Genetic resources collections, such as maintained by CGN, are rich sources of promising materials to achieve this goal.

## Electronic supplementary material

Below is the link to the electronic supplementary material.

Supplementary Figure 1 (PDF 452 KB)

Supplementary Table 1 (PDF 74 KB)

Supplementary Table 2 (XLSX 2926 KB)

## Data Availability

The metadata generated in this study are provided as supplementary material to this paper. These data are also available via CGN’s website (CGN 2017b) and via the MetaboLights data repository (http://www.ebi.ac.uk/metabolights/).
