# Supplementary material for: Metabolite variation in the lettuce gene pool: towards healthier crop varieties and food
Source: Metabolomics. 2018 Oct 29;14(11):146. doi: 10.1007/s11306-018-1443-8 (PMC6208706; doi:10.1007/s11306-018-1443-8)

Supplementary Figure 1: Biplot for the LCMS data with loadings indicated as arrows. Loadings for the phytochemicals specific for each of the species *L. sativa*, *L. serriola*, *L. saligna* and *L. virosa* (Fig. 3) are colour-marked.

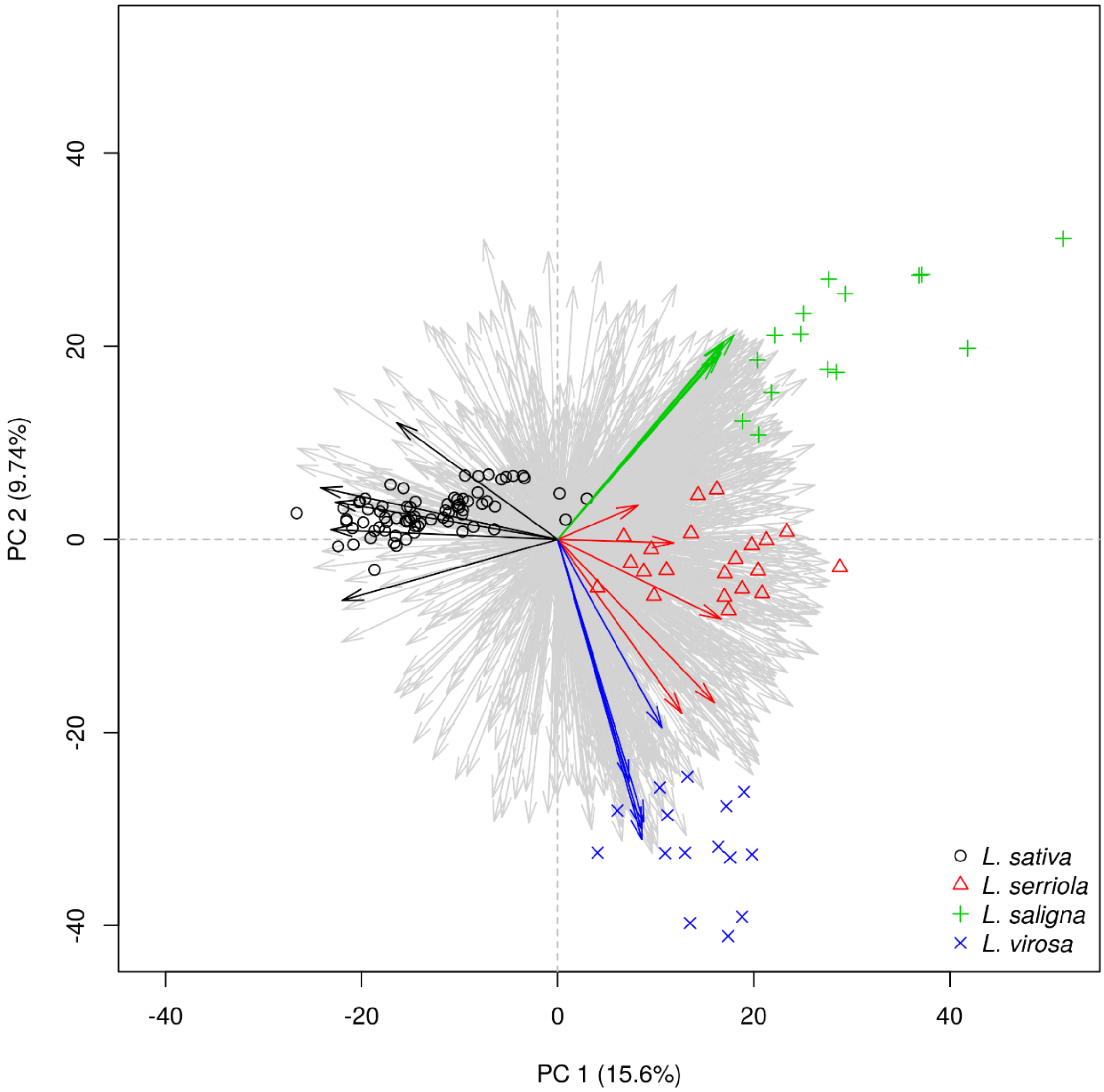

Supplement: Supplementary file 1 — Supplementary Figure 1 (PDF 452 KB) [file 11306_2018_1443_MOESM1_ESM.pdf]
