# Supplementary material for: Metabolite variation in the lettuce gene pool: towards healthier crop varieties and food
Source: Metabolomics. 2018 Oct 29;14(11):146. doi: 10.1007/s11306-018-1443-8 (PMC6208706; doi:10.1007/s11306-018-1443-8)
Supplement: Supplementary file 2 — Supplementary Table 1 (PDF 74 KB) [file 11306_2018_1443_MOESM2_ESM.pdf]

Supplementary Table 1: Origin, taxonomy and presumed gene pool status of the research lines used for metabolite profiling.

**Line**	**Source[1]**	**Species**	**Crop type**	**Gene pool**	**Line**	**Source[1]**	**Species**	**Gene pool**
TKI-006	CGN04613	*L. sativa*	Butterhead	1	TKI-150	CGN05099	*L. serriola*	1
TKI-011	CGN04818	*L. sativa*	Butterhead	1	TKI-157	CGN05153	*L. serriola*	1
TKI-016	CGN04895	*L. sativa*	Butterhead	1	TKI-160	CGN05158	*L. serriola*	1
TKI-019	CGN05140	*L. sativa*	Butterhead	1	TKI-184	CGN09309	*L. serriola*	1
TKI-020	CGN05163	*L. sativa*	Butterhead	1	TKI-194	CGN10907	*L. serriola*	1
TKI-026	CGN05813	*L. sativa*	Butterhead	1	TKI-195	CGN10938	*L. serriola*	1
TKI-036	CGN09380	*L. sativa*	Butterhead	1	TKI-197	CGN10978	*L. serriola*	1
TKI-041	CGN10966	*L. sativa*	Butterhead	1	TKI-202	CGN11334	*L. serriola*	1
TKI-042	CGN11338	*L. sativa*	Butterhead	1	TKI-204	CGN11402	*L. serriola*	1
TKI-043	CGN11340	*L. sativa*	Butterhead	1	TKI-222	CGN14263	*L. serriola*	1
TKI-045	CGN11364	*L. sativa*	Butterhead	1	TKI-232	CGN15673	*L. serriola*	1
TKI-048	CGN11403	*L. sativa*	Butterhead	1	TKI-234	CGN15678	*L. serriola*	1
TKI-052	CGN14317	*L. sativa*	Butterhead	1	TKI-252	CGN15730	*L. serriola*	1
TKI-055	CGN14640	*L. sativa*	Butterhead	1	TKI-257	CGN15737	*L. serriola*	1
TKI-061	CGN19028	*L. sativa*	Butterhead	1	TKI-260	CGN16210	*L. serriola*	1
TKI-063	CGN20139	*L. sativa*	Butterhead	1	TKI-266	CGN17389	*L. serriola*	1
TKI-064	CGN20716	*L. sativa*	Butterhead	1	TKI-275	CGN18657	*L. serriola*	1
TKI-065	CGN24530	*L. sativa*	Butterhead	1	TKI-286	CGN19052	*L. serriola*	1
TKI-066	CGN24542	*L. sativa*	Butterhead	1	TKI-338	CGN24779	*L. serriola*	1
TKI-067	CGN24683	*L. sativa*	Butterhead	1	TKI-339	CGN24780	*L. serriola*	1
TKI-069	CGN04508	*L. sativa*	Crisp	1	TKI-340	UC Davis	*L. serriola*	1
TKI-070	CGN04542	*L. sativa*	Crisp	1	TKI-342	CGN05271	*L. saligna*	2
TKI-071	CGN04581	*L. sativa*	Crisp	1	TKI-343	CGN05282	*L. saligna*	2
TKI-077	CGN05170	*L. sativa*	Crisp	1	TKI-344	CGN05301	*L. saligna*	2
TKI-078	CGN05182	*L. sativa*	Crisp	1	TKI-345	CGN05304	*L. saligna*	2
TKI-079	CGN05253	*L. sativa*	Crisp	1	TKI-355	CGN05318	*L. saligna*	2
TKI-081	CGN09381	*L. sativa*	Crisp	1	TKI-364	CGN05327	*L. saligna*	2
TKI-082	CGN10976	*L. sativa*	Crisp	1	TKI-366	CGN05330	*L. saligna*	2
TKI-083	CGN11440	*L. sativa*	Crisp	1	TKI-369	CGN05947	*L. saligna*	2
TKI-085	CGN14702	*L. sativa*	Crisp	1	TKI-374	CGN13326	*L. saligna*	2
TKI-088	CGN24676	*L. sativa*	Crisp	1	TKI-376	CGN13330	*L. saligna*	2
TKI-089	CGN24689	*L. sativa*	Crisp	1	TKI-379	CGN13375	*L. saligna*	2
TKI-090	CGN25047	*L. sativa*	Crisp	1	TKI-382	CGN15705	*L. saligna*	2
TKI-091	CGN25055	*L. sativa*	Crisp	1	TKI-383	CGN15716	*L. saligna*	2
TKI-092	UC Davis	*L. sativa*	Crisp	1	TKI-391	CGN19047	*L. saligna*	2
TKI-093	CGN06018	*L. sativa*	Latin	1	TKI-395	CGN20697	*L. saligna*	2
TKI-094	CGN09377	*L. sativa*	Latin	1	TKI-404	CGN04683	*L. virosa*	3
TKI-095	CGN11339	*L. sativa*	Latin	1	TKI-406	CGN04955	*L. virosa*	3
TKI-096	CGN13385	*L. sativa*	Latin	1	TKI-407	CGN05077	*L. virosa*	3
TKI-097	CGN24517	*L. sativa*	Latin	1	TKI-409	CGN05148	*L. virosa*	3
TKI-099	CGN04740	*L. sativa*	Cos	1	TKI-410	CGN05268	*L. virosa*	3
TKI-100	CGN04746	*L. sativa*	Cos	1	TKI-412	CGN05332	*L. virosa*	3
TKI-101	CGN04766	*L. sativa*	Cos	1	TKI-414	CGN05794	*L. virosa*	3
TKI-104	CGN05004	*L. sativa*	Cos	1	TKI-419	CGN09365	*L. virosa*	3
TKI-105	CGN05057	*L. sativa*	Cos	1	TKI-420	CGN13302	*L. virosa*	3
TKI-106	CGN05238	*L. sativa*	Cos	1	TKI-422	CGN13337	*L. virosa*	3
TKI-107	CGN05249	*L. sativa*	Cos	1	TKI-425	CGN13356	*L. virosa*	3
TKI-108	CGN05827	*L. sativa*	Cos	1	TKI-427	CGN14290	*L. virosa*	3
TKI-109	CGN06003	*L. sativa*	Cos	1	TKI-431	CGN16267	*L. virosa*	3

TKI-110	CGN09375	*L. sativa*	Cos	1	TKI-434	CGN16285	*L. virosa*	3
TKI-113	CGN14656	*L. sativa*	Cos	1	TKI-442	CGN18980	*L. virosa*	3
TKI-114	CGN16252	*L. sativa*	Cos	1	TKI-453	CGN15677	*L. georgica*	1
TKI-115	CGN18720	*L. sativa*	Cos	1	TKI-454	CGN15679	*L. georgica*	1
TKI-116	CGN24515	*L. sativa*	Cos	1	TKI-456	CGN15681	*L. georgica*	1
TKI-117	CGN24776	*L. sativa*	Cos	1	TKI-460	CGN16201	*L. georgica*	1
TKI-118	CGN04642	*L. sativa*	Cutting	1	TKI-464	CGN09357	*L. aculeata*	1
TKI-119	CGN04797	*L. sativa*	Cutting	1	TKI-465	CGN15692	*L. aculeata*	1
TKI-120	CGN05063	*L. sativa*	Cutting	1	TKI-466	CGN04664	*L. altaica*	1
TKI-121	CGN05241	*L. sativa*	Cutting	1	TKI-467	CGN15711	*L. altaica*	1
TKI-122	CGN05852	*L. sativa*	Cutting	1	TKI-468	CGN04790	*L. dregeana*	1
TKI-123	CGN10956	*L. sativa*	Cutting	1	TKI-469	CGN05805	*L. dregeana*	1
TKI-125	CGN11444	*L. sativa*	Cutting	1	TKI-471	CGN13305	*L. biennis*	
TKI-126	CGN11445	*L. sativa*	Cutting	1	TKI-472	CGN18998	*L. biennis*	
TKI-127	CGN13298	*L. sativa*	Cutting	1	TKI-473	CGN14308	*L. canadensis*	
TKI-129	CGN13367	*L. sativa*	Cutting	1	TKI-474	CGN17388	*L. canadensis*	
TKI-130	CGN14606	*L. sativa*	Cutting	1	TKI-477	CGN14312	*L. indica*	
TKI-132	CGN19069	*L. sativa*	Cutting	1	TKI-478	CGN14316	*L. indica*	
TKI-133	CGN24721	*L. sativa*	Cutting	1	TKI-483	NC097273	*L. taraxacifolia*	3
TKI-134	CGN24778	*L. sativa*	Cutting	1	TKI-484	09H5801132	*L. taraxacifolia*	3
TKI-135	CGN25052	*L. sativa*	Cutting	1	TKI-485	CGN09323	*L. perennis*	
TKI-136	CGN05061	*L. sativa*	Stalk	1	TKI-486	CGN13299	*L. perennis*	
TKI-137	CGN05292	*L. sativa*	Stalk	1	TKI-488	CGN09390	*L. tatarica*	3
TKI-138	CGN10931	*L. sativa*	Stalk	1	TKI-489	CGN18616	*L. tatarica*	3
TKI-139	CGN04777	*L. sativa*	Oilseed	1	TKI-492	CGN14301	*L. viminea*	3
TKI-140	CGN04770	*L. serriola*	Oilseed	1	TKI-493	CGN16202	*L. viminea*	3

[1] CGN: Centre for Genetic Resources, the Netherlands; UC Davis: Genome Centre, University of California, Davis, USA; NC097273: Centro de Investigación y Tecnología Agroalimentaria, Zaragoza, Spain; 09H5801132: Research Institute of Crop Production, Olomouc, Czech Republic
